# Ankylosing spondylitis diagnosis in US patients with back pain: identifying providers involved and factors associated with rheumatology referral delay

**DOI:** 10.1007/s10067-016-3231-z

**Published:** 2016-03-18

**Authors:** Atul Deodhar, Manish Mittal, Patrick Reilly, Yanjun Bao, Shivaji Manthena, Jaclyn Anderson, Avani Joshi

**Affiliations:** Oregon Health & Science University, Portland, OR USA; AbbVie Inc., North Chicago, IL USA; Division of Arthritis & Rheumatic Diseases (OP09), Oregon Health & Science University, 3181 SW Sam Jackson Park Road, Portland, OR 97239 USA

**Keywords:** Ankylosing spondylitis, Anti-TNF, Diagnostic delay, Referral strategies, Treatment patterns

## Abstract

This study aimed to identify providers involved in diagnosing ankylosing spondylitis (AS) following back pain diagnosis in the USA and to identify factors leading to the delay in rheumatology referrals. The Truven Health MarketScan® US Commercial Database was searched for patients aged 18–64 years with back pain diagnosis in a non-rheumatology setting followed by AS diagnosis in any setting during January 2000–December 2012. Patients with a rheumatologist visit on or before AS diagnosis were considered referred. Cox regression was used to determine factors associated with referral time after adjusting for age, sex, comorbidities, physician specialty, drug therapy, and imaging procedures. Of 3336 patients included, 1244 (37 %) were referred to and diagnosed by rheumatologists; the others were diagnosed in primary care (25.7 %), chiropractic/physical therapy (7 %), orthopedic surgery (3.8 %), pain clinic (3.6 %), acute care (3.4 %), and other (19.2 %) settings. Median time from back pain diagnosis to rheumatology referral was 307 days and from first rheumatologist visit to AS diagnosis was 28 days. Referred patients were more likely to be younger (hazard ratio [HR] = 0.986; *p* < 0.0001), male (HR = 1.15; *p* = 0.0163), diagnosed with uveitis (HR = 1.49; *p* = 0.0050), referred by primary care physicians (HR = 1.96; *p* < 0.0001), prescribed non-steroidal anti-inflammatory drugs (HR = 1.55; *p* < 0.0001), disease-modifying antirheumatic drugs (HR = 1.33; *p* < 0.0001), and tumor necrosis factor inhibitors (HR = 1.40; *p* = 0.0036), and to have had spinal/pelvic X-ray prior to referral (HR = 1.28; *p* = 0.0003). During 2000–2012, most patients with AS were diagnosed outside of rheumatology practices. The delay before referral to rheumatology was 10 months; AS diagnosis generally followed within a month. Earlier referral of patients with AS signs and symptoms may lead to more timely diagnosis and appropriate treatment.

**Electronic supplementary material:**

The online version of this article (doi:10.1007/s10067-016-3231-z) contains supplementary material, which is available to authorized users.

## Introduction

The delay between symptom onset and diagnosis of ankylosing spondylitis (AS) has been estimated at approximately 8 to 11 years in Europe [1–3] and approximately 13 years in the USA [4]. Diagnostic delay in AS has been attributed to the common occurrence of mechanical back pain in the general population, the typically insidious onset of the disease, young age at onset, and a lack of clinical symptoms, signs, or biomarkers unique to AS [5–7]. AS is associated with considerable pain and stiffness, impaired health-related quality of life (HRQoL), decreased work productivity, and substantial disability [5, 8]. Nonsteroidal anti-inflammatory drugs (NSAIDs) and anti-tumor necrosis factor α (anti-TNF) agents are effective in reducing pain and stiffness and improving physical function [9, 10] and are recommended in the treatment of AS [11]. Moreover, these treatments may be more effective early in the course of disease, when inflammatory processes are predominant [6, 7]. Delayed diagnosis and treatment contribute to the considerable physical, psychological, and economic burden on AS patients and their caregivers [5].

In the USA, the majority (approximately 60 %) of patients with low back pain consult with general practitioners, whereas 35–37 and 24–30 % of patients seek care from the orthopedists and chiropractors, respectively [12, 13]. However, US primary care guidelines do not explicitly specify referral to a rheumatologist in cases of suspected AS [14, 15], and it is unclear how frequently and accurately patients are diagnosed within the primary care setting.

Strategies for appropriate and timely referral to rheumatologists aimed at shortening the diagnostic delay in AS have been described [6, 7, 16]. These strategies work through improved education of health care providers and recognizing clues for better identification of possible AS patients. Because chronic back pain is often the first symptom of AS, these strategies aim to assist primary care physicians (PCPs) in distinguishing patients with inflammatory back pain from those with mechanical or nonspecific back pain and to recognize other typical clinical features of spondyloarthritis. However, specific factors that influence diagnostic and referral patterns within the primary care setting remain largely uncharacterized. To date, no studies have examined the relationship between diagnostic delay and the type of care received by AS patients prior to diagnosis.

The current study sought to identify the health care providers who make the diagnosis of AS in patients with chronic back pain in the USA, to assess treatment and referral patterns, and to identify factors associated with diagnostic delay in a large sample of patients who initially presented in non-rheumatology settings including primary care. In particular, we aimed to describe patterns of prescription drug therapy, use of diagnostic imaging, and rheumatology referral during the period from back pain diagnosis to AS diagnosis.

## Patients and methods

### Study design and patient population

**Fig. 1:**
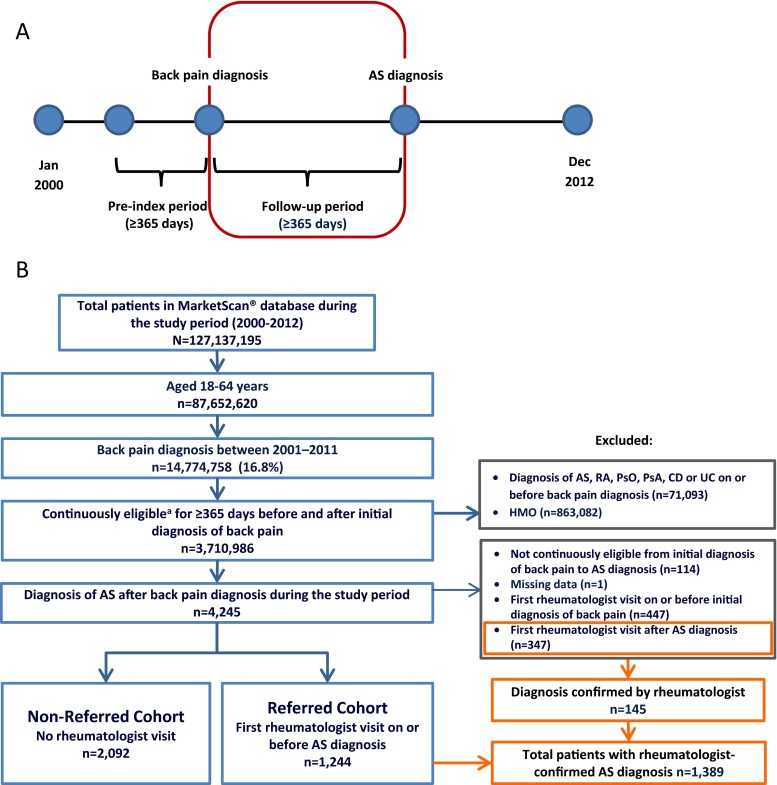
Study design and patient selection. An overview of the study design is shown in panel **a**. The follow-up period (i.e., the period of time from back pain diagnosis to AS diagnosis) is outlined in *red*. Patient flow is depicted in panel **b**. Populations included and excluded from the main analysis are depicted by the *blue* and *gray boxes*, respectively. The *orange boxes* depict a patient population that was excluded from the main analysis owing to primary diagnosis by a non-rheumatologist but who had their diagnosis subsequently confirmed by a rheumatologist. *AS* = ankylosing spondylitis; *CD* = Crohn’s disease; *HMO* = health maintenance organization; *PsA* = psoriatic arthritis; *PsO* = psoriasis; *RA* = rheumatoid arthritis; *UC* = ulcerative colitis. ^a^Patients with no interruption in insurance status

A retrospective, longitudinal cohort study was conducted using Truven Health MarketScan® US Commercial Claims Database. Pharmacy and medical claims associated with 127,137,195 patients were assessed for the January 2000–December 2012 time period. We identified patients aged 18–64 years who had an initial diagnosis of back pain in a non-rheumatology setting, followed by ≥1 diagnosis code for AS (*International Statistical Classification of Diseases*, Ninth Revision, Clinical Modification [ICD-9] code = 720.0) in any clinical setting (rheumatology or non-rheumatology; Fig. 1). Diagnosis of back pain was based on a set of 38 ICD-9 codes as described by Cherkin and colleagues [17]. The set includes diagnoses of back pain arising from a variety of non-inflammatory or mechanical etiologies, including spondylosis, spinal stenosis, spondylolisthesis, lordosis, sprains and fractures, sciatica, and others (Table S1). Continuous eligibility for ≥365 days before and after the initial back pain diagnosis date was required, and patients were followed continuously until diagnosis of AS (Fig. 1a). Individuals enrolled in health maintenance organizations were excluded, as were those with a diagnosis of chronic inflammatory diseases (AS, rheumatoid arthritis [RA], psoriasis, psoriatic arthritis, Crohn’s disease, or ulcerative colitis) on or before the initial back pain diagnosis date. Patients who had an initial rheumatologist visit before the back pain diagnosis date were also excluded. Patients who had an initial rheumatologist visit after AS diagnosis were excluded from the primary analysis; however, these patients were assessed for rheumatologist confirmation of the AS diagnosis. Patients with a rheumatologist visit on or before AS diagnosis were considered to have been referred to the rheumatologist for diagnosis (Fig. 1b; referred cohort); those with no rheumatologist visit were considered non-referred (Fig. 1b; non-referred cohort). This research was conducted in accordance with the Helsinki Declaration; exemption from Institutional Review Board review was granted because the study used de-identified data to protect patient confidentiality.

### Study measures

The study assessed the proportion of patients referred to rheumatologists for diagnosis of AS, drug prescriptions, and diagnostic imaging procedures ordered by non-rheumatologists in the period from back pain diagnosis to either AS diagnosis (for non-referred patients) or rheumatologist referral (for referred patients) and prescribing/referring physician specialty. The outcome of interest was time to referral, defined as time from the first non-rheumatologist visit for back pain to the first rheumatologist visit, at or before the time of AS diagnosis. This study included time-independent (those that could not randomly change with time) and time-dependent (those that could randomly change with time) factors that could influence the time to rheumatologist referral in AS patients. Age, sex, and comorbidities as assessed at back pain diagnosis date were considered time-independent variables. Comorbidities assessed included diabetes mellitus, cardiovascular disease, hypertension, renal disease, cancer (any), and uveitis. Time-dependent variables were captured from index date to AS diagnosis date and included physician specialty, prescription drug therapy, and diagnostic imaging procedures carried out. Physician specialty under which all prescription drug and health care service claims were provided included primary care, orthopedic surgery, pain management, chiropractic/physical therapy, acute care, and “other” (which included any specialties not specified above, as well as instances where physician information was missing). Prescription drugs assessed included NSAIDs, disease-modifying antirheumatic drug (DMARDs), corticosteroids, opiate pain medications, and anti-TNF therapy. Diagnostic imaging procedures assessed included X-rays, magnetic resonance imaging (MRI), and computed tomography (CT) scans of the spine and pelvis.

### Statistical analysis

Demographic and clinical characteristics, patterns of prescription drug use, and diagnostic imaging procedures received were compared between referred and non-referred patients using descriptive statistics. A time-dependent Cox proportional hazard model was used to determine factors associated with time to referral. The model was adjusted for age, sex, comorbidities, physician specialty, drug therapy, and imaging procedures; time-dependent variables were adjusted annually in the model. A stepwise selection method was used to determine statistically significant predictors of referral time. Hazard ratios with 95 % confidence intervals (CI) were reported. Data management and analysis was accomplished via PC-SAS® version 9.2 (SAS Institute Inc., Cary, NC), with an a priori alpha set at *p* < 0.05.

## Results

### Sociodemographic characteristics

**Fig. 2:**
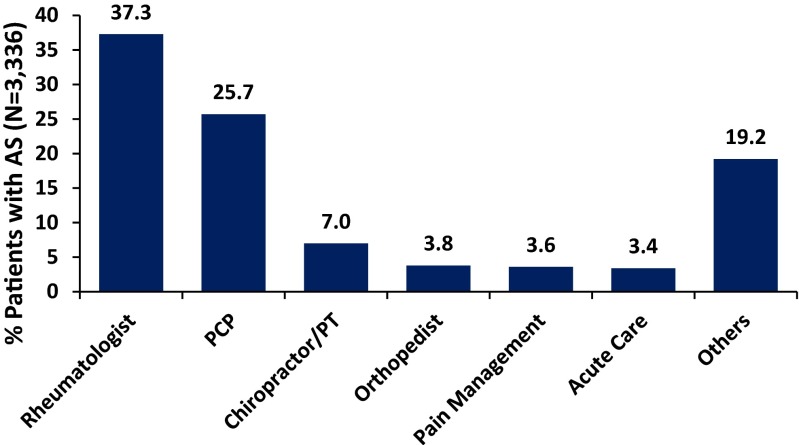
Diagnosis of AS by physician specialty. “Others” consists of any provider not specified as rheumatologist, primary care provider (*PCP*), chiropractor/physical therapist (*PT*), orthopedist, pain management, or acute care specialist or where provider specialty was missing. *AS* = ankylosing spondylitis

A total of 3336 patients had an initial diagnosis of back pain in a non-rheumatology setting, followed by a diagnosis of AS and met all inclusion criteria. Of these, 1244 (37 %) patients were referred to rheumatologists for AS diagnosis and the remaining (*n* = 2092; 63 %) were diagnosed with AS outside of rheumatology practices (Fig. 1b). Non-referred patients were most frequently diagnosed in a primary care setting (25.7 %); others were diagnosed in a chiropractic/physical therapy (7 %), orthopedic surgery (3.8 %), pain clinic (3.6 %), acute care (3.4 %), or other (19.2 %) setting (Fig. 2). An additional 347 patients were initially diagnosed by a non-rheumatologist but had a rheumatologist visit after diagnosis (Fig. 1b). Of these, 145 (41.8 %) had their AS diagnosis confirmed by the rheumatologist. The remaining 202 patients (58.2 %) were diagnosed by the rheumatologist with other disorders, including joint effusion, unspecified back disorder, rheumatoid arthritis, rheumatism not otherwise specified, osteoarthritis, and spondylosis. In total, 1389 (1244 + 145, 41.6 % of total) patients had a rheumatologist-confirmed diagnosis of AS.

A comparison of demographic and clinical characteristics between the 1244 referred and 2092 non-referred patients at the index date is shown in Table 1. Referred patients were slightly younger than non-referred patients (mean age 43 vs 46 years; *p* < 0.0001); approximately half of patients in each group were women.

**Table 1:** Patient characteristics at back pain diagnosis date

Characteristic	Patients referred to rheumatologist (*n* = 1244)	Patients not referred to rheumatologist (*n* = 2092)	*P* value^a^
Age, years	42.9	45.8	<0.0001
Female, %	50.7 %	50.0 %	0.7058
Comorbid condition, %			
Diabetes mellitus	5.1 %	9.8 %	<0.0001
Cardiovascular disease^b^	7.1 %	10.2 %	0.0025
Hypertension	18.4 %	23.5 %	0.0006
Renal disease	0.5 %	1.1 %	0.0492
Cancer (any)	17.3 %	19.0 %	0.2215
Uveitis	4.3 %	3.9 %	0.5805

^a^Chi-square test

^b^Includes myocardial infarction, ischemic heart disease, angina, cerebrovascular disease, atherosclerosis, aortic aneurysm, peripheral vascular disease, coronary artery bypass grafting, angioplasty, catheterization, and heart stenting

### Patterns of prescription drug use and imaging procedures received by referred and non-referred patients between back pain diagnosis and AS diagnosis

**Fig. 3:**
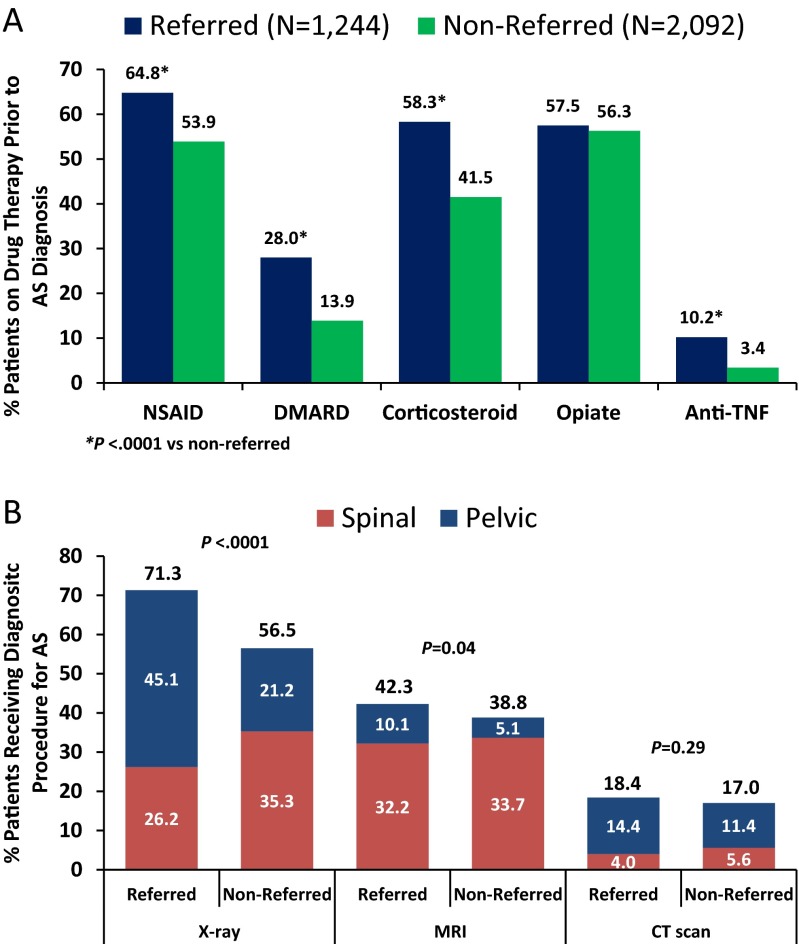
Patterns of prescription drug use (**a**) and imaging procedures (**b**) by referred and non-referred patients. *CT* = computed tomography; *DMARD* = disease-modifying antirheumatic drug; *MRI* = magnetic resonance imaging; *NSAID* = non-steroidal anti-inflammatory drug; *TNF* = tumor necrosis factor

Patterns of prescription drug use and diagnostic imaging procedures ordered by non-rheumatology providers in the period between back pain diagnosis and AS diagnosis (for 2092 non-referred patients) or rheumatologist referral (for 1244 referred patients) are summarized in Fig. 3 and Table 2. A majority of both referred and non-referred patients were prescribed NSAIDs (64.8 and 53.9 %, respectively) and opiate pain medications (57.5 and 56.3 %, respectively) (Fig. 3a). In contrast, DMARDs and anti-TNF agents were prescribed less commonly by non-rheumatology providers; anti-TNF agents were prescribed in 127 (10.2 %) of referred and 71 (3.4 %) of non-referred patients (5.9 % of total). Anti-TNF agents prescribed by non-rheumatologists were used for both inflammatory and non-inflammatory disorders diagnosed after the initial back pain diagnosis date, including RA (26.9 %), back disorder not specified (18.2 %), spondylosis (18.2 %), rheumatism not otherwise specified (15.2 %), joint effusion (15.2 %), and osteoarthritis (9.1 %). Referred patients were more likely than non-referred patients to receive prescriptions of NSAIDs, DMARDs, corticosteroids, and anti-TNF agents (*p* < 0.0001 each) prior to AS diagnosis or rheumatologist referral. As shown in Table 2, PCPs were the primary prescribers for all medications.

**Table 2:** Percentage of patients who received prescription drug therapy and imaging procedures as a function of prescribing physician specialty

	PCP (%)	Orthopedist (%)	Pain management (%)	Chiropractor/PT (%)	Acute care (%)	Other/missing (%)
Referred cohort^a^						
Prescription drugs						
NSAIDs	46.2	19.0	3.2	4.4	7.8	19.4
DMARDs	47.3	8.3	4.4	5.4	8.3	26.3
Corticosteroids	58.1	13.1	3.7	5.6	5.6	13.8
Opiates	49.4	22.6	6.4	3.8	6.4	11.4
Anti-TNF^c^	40.0	12.0	1.3	5.3	8.0	33.3
Imaging						
X-ray	22.5	12.0	2.6	8.3	22.9	31.7
CT scan	28.4	5.2	3.1	3.9	45.9	13.5
MRI	25.7	13.1	7.4	7.0	28.1	18.6
Non-referred cohort^b^						
Prescription drugs						
NSAIDs	55.4	16.6	4.5	5.7	3.1	14.7
DMARDs	65.1	8.1	2.7	3.4	5.4	15.3
Corticosteroids	62.3	12.1	3.6	2.5	5.8	13.7
Opiates	51.9	21.7	6.4	3.6	4.0	12.4
Anti-TNF^c^	59.5	13.5	0.0	2.7	2.7	21.6
Imaging						
X-ray	39.5	17.5	4.6	11.4	14.2	12.8
CT scan	31.0	6.2	6.8	2.5	37.5	16.0
MRI	32.6	10.0	6.7	6.8	25.9	18.0

Analysis includes all patients who received a given therapy or procedure (total of 100 % for each category)

*CT* computed tomography, *DMARDs* disease-modifying antirheumatic drugs, *MRI* magnetic resonance imaging, *NSAIDs* non-steroidal anti-inflammatory drugs, *PT* physical therapist, *TNF* tumor necrosis factor

^a^For the referred cohort, data represent the year prior to rheumatologist referral

^b^For the non-referred cohort, data represent the year prior to AS diagnosis

^c^Anti-TNF use was reported in 5.9 % of the total population

Approximately 75 % (*n* = 2450) of all patients received at least one diagnostic imaging procedure (X-ray, CT scan, or MRI) of the spine and/or pelvis in the period between back pain diagnosis and either AS diagnosis or rheumatologist referral (Fig. 3b). Significantly greater proportions of referred patients than non-referred patients received X-rays (71.3 vs 56.5 %; *p* < 0.0001) and MRI (42.3 vs 38.8 %; *p* = 0.0422). Referred patients were more likely than non-referred patients to have received imaging procedures specific to the pelvis (Fig. 3b). Imaging procedures (both pelvic and spinal) were primarily ordered by PCPs and acute care specialists in both referred and non-referred patients (Table 2).

### Factors associated with the time to rheumatologist referral

The median time from back pain diagnosis to rheumatologist referral was 307 (interquartile range 81–782) days, and median time from referral to AS diagnosis was 28 (interquartile range 0–194) days. Factors associated with referral time are presented in Table 3. Over the study period, the probability of referral decreased by approximately 1 % for each year of age, was 15 % greater among men versus women, and was 49 % greater among patients with uveitis versus those without uveitis. Other comorbidities were not significantly associated with referral time. With the exception of corticosteroids, prescription drug usage during the time from back pain diagnosis to AS diagnosis was strongly associated with referral time; the probability of referral was 55 % greater among patients who received NSAIDs, 33 % greater among patients who received DMARDs, 40 % greater among patients who received anti-TNF therapy, and 18 % lower among patients who received opiates. Patients who received X-rays (pelvic and/or spinal) during the period from back pain diagnosis to AS diagnosis were 28 % more likely to be referred than those without X-ray, whereas patients with CT scans (pelvic and/or spinal) were 29 % less likely to be referred. MRI was not significantly associated with referral time.

**Table 3:** Factors associated with rheumatologist referral time for patients with ankylosing spondylitis in multivariate analysis

Predictor^a^	HR (95 % CI)	*P* value
Age	0.986 (0.981, 0.991)	<0.0001
Sex (male vs female)	1.15 (1.03, 1.29)	0.0163
Uveitis	1.49 (1.13, 1.96)	0.0050
Specialty		
PCP	1.96 (1.64, 2.35)	<0.0001
Pain management	0.79 (0.69, 0.91)	0.0013
Prescribed drug therapy		
NSAIDs	1.55 (1.35, 1.77)	<0.0001
DMARDs	1.33 (1.16, 1.54)	<0.0001
Opiate	0.82 (0.72, 0.94)	0.0048
Anti-TNF	1.40 (1.12, 1.76)	0.0036
Spinal/pelvic imaging procedure		
X-ray	1.28 (1.12, 1.46)	0.0003
CT scan	0.71 (0.58, 0.87)	0.0009

^a^Only statistically significant predictors are presented

*CI* confidence interval, *CT* computed tomography, *DMARDs* disease-modifying antirheumatic drugs, *HR* hazard ratio, *NSAID* non-steroidal anti-inflammatory drugs, *PCP* primary care physician, *TNF* tumor necrosis factor

## Discussion

This large, retrospective analysis of the administrative claims data of 127 million individuals over a 10-year period provides several unique observations regarding the diagnosis of AS in the USA. First, of all patients with an initial diagnosis of back pain who subsequently were diagnosed with AS, only 37 % were referred to rheumatologists. The remaining 63 % were diagnosed by non-rheumatologists. Previous studies have shown that non-rheumatology providers see most patients with chronic back pain. For example, an analysis of data from the National Health and Nutrition Examination Survey (NHANES 1976–1980) showed that patients with low back pain most commonly sought care from PCPs (approximately 60 %), followed by orthopedists (30 %) and chiropractors (30 %) [12]. Similar results were reported in a 2006 survey of 2809 patients with back and/or neck pain in North Carolina [13]. In addition, in a recent survey of 190 rheumatologists in the USA and Canada, the majority (95 %) of rheumatologists reported that PCPs were the main referral source for patients with chronic back pain beginning before age 45, followed by physical/occupational therapists (24 %), chiropractors (18 %), and other specialists (orthopedists, pain management specialists, and psychiatrists [28 %]) [18]. Another recent study suggested that <10 % of patients with AS in the USA self-refer to rheumatology [4]. It was surprising, however, that as many as two thirds of patients remained in the non-rheumatology setting for diagnosis of AS. Although the basis for the low rate of referral is unclear, it may be related to the lack of accessibility and/or long waiting times for rheumatology care as well as a focus on appropriate surgical referral in US-specific primary care guidelines with little guidance on when to consider rheumatology referral [14, 15, 19–21].

Second, the median delay from back pain diagnosis to rheumatologist referral was approximately 10 months, but following consultation with a rheumatologist, patients were generally diagnosed with AS within 1 month. Predictors of shorter time to referral included younger age, male sex, presence of uveitis, increased use of prescription drug therapy, use of X-ray imaging, and PCP as the referring physician specialty. These results suggest that referral decisions may be driven both by appropriate recognition of AS features (young age of onset, uveitis, and structural damage) and by a continued misperception among non-rheumatologists that AS is rarely seen in women [22]. Increased use of prescription drug therapy was also associated with shorter time to referral, which may suggest that referred patients had greater disease activity than non-referred patients. Although PCPs were more likely than other specialists to refer in a timely manner, our results suggest that better identification and earlier referral would be associated with faster and more accurate diagnosis, supporting the use of referral strategies in the primary care setting [6].

Third, of patients who were initially diagnosed with AS by a non-rheumatologist and then referred to a rheumatologist, only 42 % were confirmed to have AS, and the diagnosis was changed in 58 % of the patients—mostly to a non-inflammatory condition—by the rheumatologists. Among many other possible reasons, inappropriate use of imaging modalities by non-rheumatologists may have contributed to misdiagnosis. In particular, only 21 % of non-referred and 45 % of referred patients received pelvic X-rays by non-rheumatologists, which are essential for the diagnosis of AS [23]. MRI of the pelvis was rarely performed in the non-rheumatology setting, though MRI of the spine was ordered by non-rheumatologists in approximately one third of both referred and non-referred patients, highlighting an educational gap among non-rheumatologists in the understanding of appropriate imaging tests when considering an AS diagnosis. In addition, approximately 6 % of the patients were prescribed anti-TNF agents by non-rheumatologists without confirmation of an immune-mediated inflammatory disorder. This observation, combined with the high rate of misdiagnosis of AS, raises the issue of potential inappropriate use of expensive and potentially hazardous medications by non-specialists.

Our results are generally consistent with previous studies showing insufficient awareness of AS in the primary care setting. In a study of 807 patients with AS in the UK, the mean diagnostic delay was 8.6 years, yet a majority of patients (62.1 %) reported consulting a health care practitioner within 1 year of symptom onset. Because most of these patients were ultimately referred to rheumatologists, the authors suggested that the diagnostic delay was associated with inadequate recognition of signs and symptoms by primary care physicians [21]. In another study of 70 patients with AS in India, incorrect initial diagnoses were made in 77 % of patients. Misdiagnoses were most frequently ascribed to orthopedists and primary care physicians, and the authors concluded that misdiagnosis was the largest contributor to diagnostic delay in their sample [24].

One of the strengths of our study is the use of a large administrative claims database, which allowed the assessment of real-world pharmacy and medical claims in a large, nationally representative population of patients with AS. However, it contains some limitations inherent to claims analysis. The accuracy of the diagnosis codes used to identify patients included and excluded from the analysis is unknown; however, we note that the database used in the current study has been exploited extensively in previous analyses. In addition, we could not capture clinical factors such as disease severity and non-prescription NSAID use. In addition, our study assessed the time from back pain *diagnosis* to AS diagnosis, rather than delay between back pain *onset* to AS diagnosis. Therefore, it was not possible to compare our results directly to studies that determined diagnostic delay relative to symptom onset. Additionally, patients with chronic back pain who were not ultimately diagnosed with AS are not included in this dataset and it is not known how these patients are managed. In addition, due to limitations in ICD-9 coding, our data set included both acute and chronic back pain prior to AS diagnosis and also included patients with diagnoses of back pain arising from a variety of non-inflammatory or mechanical etiologies, including spondylosis, spinal stenosis, spondylolisthesis, lordosis, sprains and fractures, sciatica, and others (Table S1). By including a comprehensive panel of back pain diagnoses, our study captured a large and diverse patient sample, which may lead to overestimation of study results.

## Conclusions

Only one third of patients with AS with a recorded diagnosis of chronic back pain were referred to rheumatologists before a diagnosis of AS was made. The median delay from back pain diagnosis to rheumatologist referral was approximately 10 months, and following consultation with a rheumatologist, patients were generally diagnosed with AS within 1 month. Younger age, male gender, presence of uveitis, increased use of drug therapy and pelvic and/or spinal X-rays, and PCP as the referring physician were each associated with shorter time to rheumatology referral. Improved awareness of AS signs and symptoms in the primary care setting may lead to more timely and appropriate rheumatology referrals and subsequently accurate diagnosis and appropriate treatment.

## Electronic supplementary material

Below is the link to the electronic supplementary material.

ESM 1 Table S1. ICD-9 diagnoses codes associated with non-inflammatory or mechanical back pain (PDF 81 kb)
