# Supplementary material for: Ankylosing spondylitis diagnosis in US patients with back pain: identifying providers involved and factors associated with rheumatology referral delay
Source: Clin Rheumatol. 2016 Mar 18;35:1769–76. doi: 10.1007/s10067-016-3231-z (PMC4914524; doi:10.1007/s10067-016-3231-z)
Supplement: Supplementary file 1 — Table S1. ICD-9 diagnoses codes associated with non-inflammatory or mechanical back pain (PDF 81 kb) [file 10067_2016_3231_MOESM1_ESM.pdf]

SUPPLEMENTARY MATERIAL

Table S1. ICD-9 diagnoses codes associated with non-inflammatory or mechanical back pain

ICD-9 Code	Diagnosis
307.89	Psychogenic backache
719.49	Pain in Joint
721.42	Lumbar spondylosis with myelopathy
721.6	Ankylosing vertebral hyperostosis
721.7	Traumatic spondylopathy
721.8	Other allied disorders of spine
721.9	Spondylosis of unspecified site without myelopathy
721.91	Spondylosis of unspecified site with myelopathy
722.1	Displacement of lumbar disc without myelopathy
722.2	Displacement of unspecified disc without myelopathy
722.32	Intervertebral disc disorders (lumbar region)
722.52	Degeneration of lumbar or lumbosacral Intervertebral disc
722.6	Degeneration of intervertebral disc, site unspecified
722.73	Intervertebral disc disorders with myelopathy (lumbar region)
722.83	Postlaminectomy syndrome (lumbar region)

722.9	Other and unspecified disc disorder, unspecified region
722.93	Other and unspecified disc disorder, lumbar region
724	Spinal stenosis other than cervical, unspecified region
724.02	Spinal stenosis other than cervical, lumbar region
724.09	Spinal stenosis other than cervical, other
724.2	Lumbago (nonspecific backache)
724.3	Sciatica
724.4	Thoracic or lumbosacral neuritis or radiculitis, unspecified
724.5	Backache, unspecified
724.6	Disorder of sacrum
724.8	Other symptoms referable to back
724.9	Other unspecified back disorders
737.2	Lordosis
738.4	Acquired spondylolisthesis
739.3	Nonallopathic lesions, not elsewhere classified, lumbar region
739.4	Nonallopathic lesions, not elsewhere classified, sacral region
756.1	Anomaly of spine, unspecified
756.12	Spondylolisthesis
805.4	Lumbar fracture
805.6	Sacrum and coccyx fracture

805.8	Vertebral fracture of unspecified site
846.x	Sprains and strains of sacroiliac region
996.4	Mechanical complication of internal orthopedic device, implant and graft
